# Willingness to pay for health gains from an international integrated early warning system for infectious disease outbreaks

**DOI:** 10.1007/s10198-022-01527-w

**Published:** 2022-09-28

**Authors:** Meg Perry-Duxbury, Sebastian Himmler, Job van Exel, Werner Brouwer

**Affiliations:** 1 Erasmus School of Health Policy & Management, Erasmus University Rotterdam, P.O. Box 1738, 3000 DR Rotterdam, The Netherlands; 2 Erasmus Centre for Health Economics Rotterdam (EsCHER), Erasmus University Rotterdam, Rotterdam, The Netherlands

**Keywords:** Infectious disease outbreaks, Early warning system, Willingness to pay, Contingent valuation, I18, H41

## Abstract

Recently, due to the corona virus outbreak, pandemics and their effects have been at the forefront of the research agenda. However, estimates of the perceived value of early warning systems (EWSs) for identifying, containing, and mitigating outbreaks remain scarce. This paper aims to show how potential health gains due to an international EWS might be valued. This paper reports on a study into willingness to pay (WTP) in six European countries for health gains due to an EWS. The context in which health is gained, those affected, and the reduction in risk of contracting the disease generated by the EWS are varied across seven scenarios. Using linear regression, we analyse this ‘augmented’ willingness to pay for a QALY (WTP-Q) for each of the scenarios, where ‘augmented’ refers to the possible inclusion of context specific elements of value, such as feelings of safety. An initial WTP-Q estimate for the basic scenario is €17,400. This can be interpreted as a threshold for investment per QALY into an EWS. Overall, WTP estimates move in the expected directions (e.g. higher risk reduction leads to higher WTP). However, changes in respondents’ WTP for reductions in risk were not proportional to the magnitude of the change in risk reduction. This study provided estimates of the monetary value of health gains in the context of a pandemic under seven scenarios which differ in terms of outcome, risk reduction and those affected. It also highlights the importance of future research into optimal ways of eliciting thresholds for investments in public health interventions.

## Introduction

Recently, pandemics and their effects, both on health and the economy, have been at the forefront of the research agenda. The current coronavirus pandemic, along with other recent infectious outbreaks such as Ebola, SARS, and H1N1, has highlighted the importance of infectious disease control [1]. In 2015, an interdisciplinary research network that investigates the potential of an international integrated early warning system (EWS) for identifying, containing, and mitigating large infectious outbreaks was initiated by the EU [2]. Currently only 81 countries have a national strategy for disaster risk reduction (such as an EWS), few explicitly mention pandemic threats [3], and especially those of predominantly island nations (for example New Zealand, Taiwan, Singapore) have successfully mitigated the impact of COVID-19. An international EWS would upgrade existing regional systems, and would strengthen the international cooperation required to address current and future threats of infectious diseases [4]. This would be especially beneficial to nations that do share (open) borders with other countries, and may thus find the migration of a virus harder to control.

Given the excessive costs of a pandemic—the coronavirus pandemic has been estimated to cost 1–2% of global GDP so far [5]—it is only logical to want to prevent such situations, even when EWSs themselves are costly. Potential benefits of an effective warning system are clear and include a reduction in disease burden [6], increased feelings of safety [7], and a smaller negative impact on the economy [8]. However, these potential benefits are difficult to calculate as they are abstract, uncertain, and, at least partially, may occur in the far future. Additionally, if outbreaks are stopped early on, benefits of the system will not be visible to the general public, meaning that acceptance of high spending contributions to EWSs may be limited, even if the benefits far outweigh the potential costs of outbreaks. Quantifying the perceived value of these systems informs us of what level of contributions society would find acceptable.

How to elicit a value for improved (feelings of) health safety in the context of recurrent threats of pandemics was addressed in a recent review by Perry-Duxbury et al. [9]. They examined the methodologies commonly used for safety valuation across various topics within applied economics, specifically transportation, environment and health. Of the 33 papers reviewed, 22 used willingness to pay (WTP), a form of the contingent valuation method, while the other 11 used discrete choice experiments. Himmler et al. [7], building on this review, subsequently investigated individuals’ WTP for an EWS and reported a mean estimate of €21.80 (median €10.00) per household per month from a sample from six European countries—Denmark, Germany, Hungary, Italy, the Netherlands, and the UK (as measured *before* the current COVID-19 outbreak). They found that 80–90% of people would be willing to pay at least some additional tax towards an increase in health safety via an EWS [7]. Notably, these results were obtained from a survey question in which the magnitude of the health gain generated by an EWS was not explicitly mentioned or defined.

We can provide additional insights into the ‘acceptable’ level of contributions towards an EWS by exploring the value of an EWS while explicating the potential health gains it will provide. This will further allow us to calculate implied WTP per unit of health gain (such as a quality adjusted life year (QALY)). Mean WTP (for a QALY) can then be viewed as an upper-bound for spending on this preventative system, with the aggregate upper-bound being mean WTP multiplied by the number of contributing citizens. This estimate may differ from similar estimates focusing on curative interventions for several reasons. First, in the case of an unidentified pandemic, it is harder to predict who will be affected by the virus and to what extent. Second, preventative programmes such as an EWS lead to health benefits in the future (i.e. QALY gains) but potentially also to positive externalities now, such as the feeling of safety. It is, therefore, likely that WTP for an EWS does not solely consider (future) QALY gains, but also less tangible gains in (current) well-being.

This paper reports on a study into the WTP in six European countries for improved (feelings of) health safety in the context of preventing outbreaks. The study includes tests for sensitivity to scale and context using seven separate scenarios. They vary by the context in which health is gained (i.e. 1 year decrease in health or immediate death), social inclusivity, and by how much an EWS reduces the risk of contracting the disease (4% to 2%, 4% to 0%, and 60% to 20%). This is relevant as research has shown that in WTP questionnaires elicited values may vary substantially across the context in which QALYs are gained [10].

## Background

When estimating the WTP for a particular good like an EWS, and also when estimating the implied WTP per QALY (WTP-Q), there are various methodological issues to bear in mind. While some relate to the interpretation of the elicited value by the researcher, such as the definition of outliers and protest answers, there are also more conceptual concerns surrounding this approach. Anchoring—a pervasive judgement bias in human decision making—is one such issue that may systematically influence elicited WTP values [11]. While this can relate to the impact of a random value being shown before a valuation question is asked [12], in the case of contingent valuation studies it often refers to the initial bid for the first WTP question asked in the survey. This bid then works as an anchor in subsequent WTP answers.

Other relevant methodological concerns regarding WTP elicitation include sensitivity to scale and probability weighting. The probability of infection varies dramatically across different infectious diseases and is both relevant in itself and in its impact on the health gain generated by preventing the disease. Prospect theory suggests that people tend to overweight small probabilities and underweight larger ones [13]. Probability weighting, where probabilities are corrected for being treated non-linearly, is one way to address this issue. Bobinac et al. demonstrate the impact of probability weighting on WTP for a Quality Adjusted Life Year (QALY) and report improved validity of the corrected estimates [14].

Sensitivity to scale relates to whether WTP estimates adequately reflect the size of the good on offer. Even if WTP responses are ‘theoretically valid’, i.e. they increase with the size of the (health) gain offered, Bobinac et al. (2012) argue this is not a sufficient condition for the practical use or ‘theoretical plausibility’ of the estimate. For example, someone may be willing to pay 50€ for a health gain of 1 QALY but only 51€ for a health gain of 2 QALYs. While the determination of a ‘practically meaningful’ result may be arbitrary, we can test whether WTP estimates are (at least close to) proportional to the size of the gain [15]. Bobinac et al. found that WTP-Q estimates were highly insensitive to duration and type of health gain, which precludes establishing a ‘unique’ value per QALY [15].

If there is insufficient sensitivity to scale in WTP responses, the valuation of WTP-Q will be strongly influenced by the size of the health gain offered in the WTP question. This could lead to a large range of possible estimations of WTP-Q if the EWS in question were to cover a variety of infectious diseases with varying rates of infection. Acknowledging sensitivity to scale and probability weighting (along with anchoring effects), also in analysing WTP data, can enhance our understanding of the extent of uncertainty surrounding the estimates and may indicate improvements in eliciting and analysing WTP data. Given that the values elicited for health gains in the context of an EWS can inform the decision-making process, said insights will further inform investment choices for the warning system mentioned earlier.

## Methods

### Data

A contingent valuation experiment on the monetary valuation of a change in quality-of-life or life-years—asked within the context of an EWS for infectious disease—was conducted via an online survey in general population samples from six European countries: Denmark, Germany, Hungary, Italy, The Netherlands and the UK, from February to March of 2018. The survey covered citizens between the ages of 18 and 65 so as to limit the population to income taxpayers, given that tax was the payment vehicle in the questionnaire. Respondents were recruited using stratified sampling and an online sampling agency (Dynata). The samples aimed to be representative of national populations aged 18 to 65 regarding age and gender. The sample size from each country was around 500 respondents, which is common for willingness-to-pay studies in the health domain [10, 16]. The number of countries was chosen to accommodate the budget we had available for the data collection. More specific information on the data collection itself and reasoning behind the survey design can be found in Himmler et al. [7].

### Willingness-to-pay scenarios

The survey consisted of WTP questions for seven different scenarios (preceded by a warm-up question and a question in which risk was excluded; reported elsewhere (Himmler et al. [7])), which differed in the risk of infection and the outcome of infection in relation to the effectiveness of an EWS. Some scenarios were asked from the socially inclusive-personal (SIP) perspective, while others were asked from the socially exclusive perspective. Social exclusive scenarios covered situations where (1) only members of the opposite sex or (2) only children benefit from the warning system and receive the health gain. It is important to note that the social perspective, while excluding the respondent, captures elements of altruism and possible closeness to the specific situation. For example, if the respondent has children, this may affect the observed WTP. These different groupings help to provide additional insight into both valuing health gains under a societal perspective and the question of equity weighting—whether or not health gains need to be weighted based on demographic characteristics such as age. For example, Bobinac et al. found that WTP-Q values varied substantially (between €52,000 and €83,000) dependent on whether the societal perspective used includes the individual responding to the question or not [17]. Regarding the scenario in which children alone are affected, it has been shown that individuals’ attach a higher weight to treatments for relatively younger patients (and the more severely ill) [18].

**Fig. 1:**
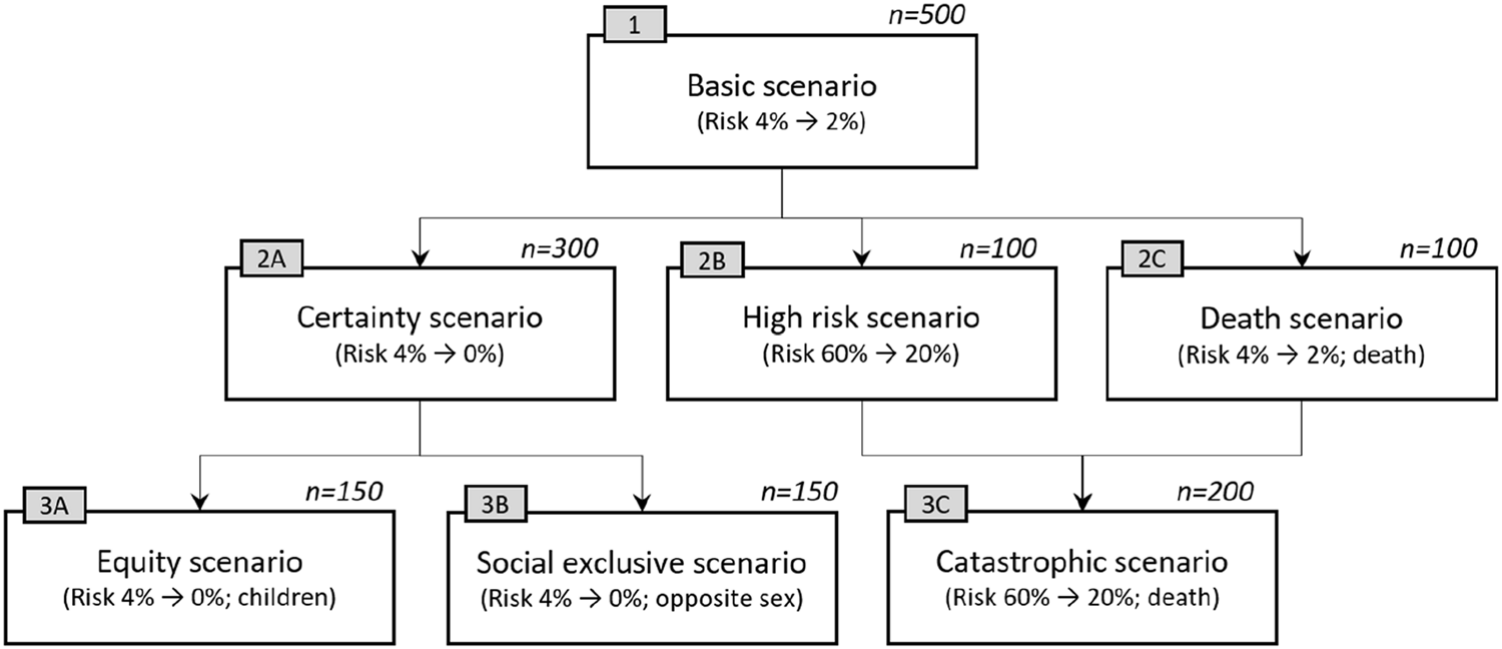
Scenarios & questionnaire flow (*n* = respondents per country)

For ease of identification, the seven scenarios are referred to as the *Basic, Certainty, High risk, Death, Equity, Social exclusive*, and *Catastrophic* scenarios, respectively, and are summarised in Fig. 1. Each respondent was presented with three scenarios; all respondents answered the basic scenario before they were randomized to either scenario 2A, 2B, or 2C, followed by either scenario 3A, 3B, or 3C according to the flow and ratios presented in Fig. 1.

The health effect of the EWS in each scenario was described by a scenario-specific risk reduction of becoming infected by a hypothetical disease. In scenarios 1, 2A, 2B, 3A, and 3B, becoming infected would cause a drop in health from ‘Health state 1’ (HS1) to ‘Health State 2’ (HS2). In scenarios 2C and 3C, an infection would lead to the death of the individual, who was in HS1 before.

### Valuation of health states used in scenarios

At the start of the questionnaire, respondents were asked to value these two health states, which were described using the descriptive system of the EQ-5D instrument, which consists of five dimensions each distinguished by five levels [19]. The corresponding tasks are shown in Appendix 2. Respondents valued the health states using a visual analogue scale (VAS) with a range of 0 (worst imaginable health) to 100 (best imaginable health). The two health states differed on all dimensions, with HS1 dominating HS2 on all dimensions. Using the EQ-5D-5L value set for England [20], the index scores for HS1 and HS2 were 0.853 and 0.524 (on a scale from 1, being in full health, to 0, being dead), respectively, and HS1 was thus expected to be valued higher than HS2. In addition to being asked to value HS1 and HS2, respondents were asked to value perfect health, death, and their current health on a VAS scale. After valuation, respondents were also explicitly asked to choose which of the two health states they thought was ‘better’.

### Willingness-to-pay question

In the WTP questions themselves, respondents were initially asked, using a payment scale, what they would definitely be willing to pay per month for a reduction in risk, with taxes as the payment vehicle. The risk reduction referred to an outcome of either 1 year in the worse health state (from the better health state) or immediate death (from the better health state). In a second step, respondents were asked to mark the amounts they would definitely not be willing to pay per month on the same payment scale. Finally, respondents had to fill in an exact amount within this interval. These exact values were used in the subsequent analysis. A complete example of a scenario description and the respective questions asked can be found in Appendix 3. After completing the three WTP scenarios, questions were asked about demographic characteristics, and awareness of infectious disease outbreaks.

### WTP data conditioning

Before analysing the data, all monetary values (i.e. WTP and income) from UK, Danish, and Hungarian samples were converted to euro values using average exchange rates during the month of sampling. Additionally, all monetary answers were adjusted for purchasing power parity (PPP) using the European 28 countries index as a base [21]. Further pre-processing of the data related to whether respondents understood the health impact in the scenarios: If respondents indicated that they would prefer HS2 over HS1 in the direct comparison (Appendix 2), their answers were removed from further analysis. This was because the health changes in the scenarios were described using these health states, and HS1 is clearly dominating HS2 in all presented dimensions. This led to the exclusion of 7% of the sample (Appendix 1, or Table 1 “data quality”). Further illogical or inconsistent answers in the tasks shown in Appendix 2 were summarised in a dummy variable and included as control in the regression analysis. Finally, protest zero answers and WTP outliers were set to missing per question. Individuals who chose 0 as their maximum WTP had to select one of the following options to specify the reason for this answer: (i) not worth more than 0, (ii) unable to pay more than 0, (iii) government task, or (iv) the option to formulate another reason in an open text field. The first two options were considered to indicate a true WTP of zero, while “government task” indicated a protest zero. Entries in the open text field were evaluated and labelled as either true zero or protest zero. WTP values were labelled as outliers when they exceeded the upper outer fence of the interquartile range (Table 1) [22, 23].

**Table 1:** True zeros, protest answers, and outliers

Scenarios	Initial observations	After excluding due to data quality	Excluded		Included	
			# Protest zeros (%)	# Outliers after protest exclusion (%)	# Observations after cleaning	# Thereof true zeros (%)
Base	3140	2920	268 (9.18)	110 (4.15)	2542	124 (4.88)
Certainty	1734	1608	154 (9.58)	52 (3.58)	1402	89 (6.35)
High risk	760	707	71 (10.04)	20 (3.14)	616	23 (3.73)
Death	646	605	37 (6.12)	26 (4.58)	542	22 (4.06)
Equity	880	814	59 (7.25)	30 (3.97)	725	34 (4.69)
Social exclusive	854	794	136 (17.13)	31 (4.71)	627	48 (7.66)
Catastrophic	1406	1312	119 (9.07)	52 (4.36)	1141	40 (3.51)

### Further data cleaning

Individuals were asked about their gross monthly household income by first prompting them to select an income range (country and currency specific). In a second step, respondents could indicate an exact amount within that range. If an exact amount was not provided, income was imputed based on the mean income of respondents, who did provide an exact amount, within the selected income range. After this, descriptive statistics were prepared to show an overview of the sample (Table 2). The mean age of the sample was 42 years old and half of the sample was male. Mean gross monthly household income was €2753 and the majority of the sample were married and employed (or self-employed). Appendix 1 shows that the analysis sample is largely representative to the population of individuals aged between 18 and 65 in terms of the age and gender distribution.

**Table 2:** Summary statistics

	Mean (sd)	Min	Max	Observations
Age	42.01 (13.98)	18	65	2920
Monthly Household Income in EUR (gross)	2753	0	10,796	2536
EQ-5D-5L score^†^	0.86 (0.12)	− 0.378	1	2920
Awareness of outbreaks	54.3 (9.72)	12	84	2920
Male	0.49 (0.50)			2920
Married	0.57 (0.49)			2920
Education				
No tertiary education	0.59 (0.49)			2920
Tertiary education	0.41 (0.49)			2920
Employment				
Employed	0.55 (0.50)			2920
Self-employed	0.10 (0.30)			2920
Unemployed	0.06 (0.24)			2920
Homemaker	0.07 (0.25)			2920
Student	0.10 (0.31)			2920
Retired	0.09 (0.28)			2920

^†^Country specific value sets were used where available. Value sets from Spain and Germany were used for Italy and Denmark, respectively)

Self-Assessed Health was measured as the EQ-5D-5L score provided by each respondent in the questionnaire. Country specific value sets were used where available. When unavailable, value sets from countries in the same region in Europe for which a value set is available were used (Spain and Germany for Italy and Denmark, respectively) [20, 24–27]. Awareness of outbreaks was assessed using 12 questions on a Likert scale from 0 to 7, with higher values indicating a higher level of awareness of the existence, impact, and likelihood of infectious diseases outbreaks. For more detail on this measure, we refer to Himmler et al. [7].

### Willingness-to-pay differences for scenarios and determinants of WTP

A model was estimated with WTP values as the outcome to see the effects of the different elements that make up the seven scenarios on WTP, along with the effects of other determinants such as age and income (Eq. (1). The elements of the scenarios considered in this question can all be traced back to Fig. 1, the elements are: whether the risk difference is large (40%) or small (4%, 2%), whether it is certain that the EWS prevents all infections (4–0%), whether the perspective is socially exclusive or SIP and whether the outcome of the disease is death or 1 year in worse health. The seven scenarios were designed by varying these elements—for example, the certainty scenario has a small risk difference, a certain drop in risk to zero percent and an outcome of 1 year in worse health. All of the aforementioned variables were treated as dummy variables. We also included a variable for the difference between better and worse health (or better health and death) to investigate whether the size of the health loss had an impact on individual WTP responses. Education, employment, and country are all categorical variables and were treated as such. Given the skewed nature of income, log of income was used for ease of interpretation. We also transformed WTP to ln(WTP + 1)—as WTP data were skewed and the plus one allowed for the inclusion of (true) zero WTP answers in the analysis.

1 $$\mathrm{ln}\left(WTP+1\right)=\,\alpha +{{\beta }_{1}LargeRisk + {\beta }_{2}CertainRisk {+ {\beta }_{3}SocialExclusive+ {\beta }_{3}OutcomeDeath+ {\beta }_{4}HealthChange+ \beta }_{5}married+ \beta }_{6}\mathrm{ln}\left(income\right)+ {\beta }_{7}age+ {\beta }_{8}{age}^{2}+ {\beta }_{9}male {+ \beta }_{10}SAH+ {\beta }_{11}education + {\beta }_{12}employment+ {\beta }_{12}awareness+ {\beta }_{13 }country+ \varepsilon$$

### Weighting, rescaling, and transforming of WTP data for WTP-Q estimation

As mentioned in previous sections, the aim of this paper was not only to assess the effect of different scenarios on WTP, but also to present WTP-Q estimates. To do so, we needed estimates for an individual’s expected health gain for each scenario. In this survey, the outcome of introducing an EWS for infectious diseases led to some reduction in risk of either (1) a loss in quality-of-life for 1 year—from the previously designated better health state to the worse health state—or (2) a large loss in life-years—from the better health state to immediate (and permanent) death. Therefore, the expected QALY gain (E(Q)) per scenario was calculated as:

2 $${E(Q)}_{scenario}=\left(Q\left({HS}_{better}\right)- Q\left({HS}_{worse or death}\right)\right)*{p}_{scenario}*LY,$$

where $$Q\left({HS}_{better}\right)- Q\left({HS}_{worse or death}\right)$$ is the quality-of-life loss from becoming infected based on the sample average VAS scores for the health states, and $${p}_{scenario}$$ represents the reduction in risk from the introduction of the warning system for the specific scenario. The sample average valuations were used instead of the individual valuations, a common approach in health state valuation, due to concerns about the validity of individual values (see Appendix 1). $$LY$$ stands for life-years, and refers to the years gained due to the reduction in risk. These varied dependent on the type of risk; in scenarios where there was only a change in QoL (for 1 year) before returning to better health, the increase in life-years was zero. However, for scenarios where death was the risk posed by the infectious disease (Death and Catastrophic scenarios) the change in QoL was permanent and covered all the remaining life-years. In the equation, *LY* represents remaining period life-expectancy conditional on age, country and gender, taken from the Human Mortality Database lifetables [28].

Given that respondents valued death and perfect health at scores other than 0 and 100 on a VAS score [29], the raw responses to HS1 and HS2 were rescaled, as recommended by and done in previous studies [15, 29], so that the endpoints of the VAS scale were effectively labelled as ‘Perfect health’ and ‘Death’ (the QALY scale), rather than their original labels of ‘best imaginable health’ and ‘worst imaginable health’ (Eq. 3).

3 $${Rescaled HS}_{\mathrm{1,2}}= \frac{Raw\,{HS}_{\mathrm{1,2}}-{HS}_{death}}{{HS}_{perfect}-{HS}_{death}}$$

Scores for health states worse than death were capped at − 1, and at 1 for health states ‘better than perfect health’ which occur when a respondent values the health state in question higher than their score for perfect health. After rescaling, the sample average differences between the health states were calculated.

In this paper, the effects of probability weighting on WTP-Q are also shown, as it has been found that adjusting for probability weighting improves the validity of WTP-Q estimates [14]. To do this, both non-weighted probabilities (i.e. those shown in the questionnaire) and weighted probabilities were used to estimate expected QALY gains. To be able to compare results with Bobinac et al. [14] we used the same three specifications of the probability weighting function: the one-parameter Tversky and Kahneman (TK) function [13], the two-parameter Gonzalez and Wu (GW) function [30], and the one-parameter Prelec (P) function [31], using the function parameters estimated by Bleichrodt and Pinto Prades for TK and P functions [32], and the parameters estimated by Abdellaoui for the GW function [33]. The weighted probabilities for the questionnaire (Appendix 1) were used for ${p}_{scenario}$ in Eq. 2.

### Estimating WTP-Q using two different approaches

Once the (probability weighted and rescaled) expected QALY estimates were calculated, we calculated the WTP-Q per scenario. We present two alternative estimation approaches. The first entailed calculating WTP-Q by dividing the scenario-specific yearly WTP by the scenario specific average expected QALY gain (Eq. 4)). This is hereafter called ‘ratio of means’. The second approach used linear regression to predict WTP-Q estimates (Eq. 5). This implied first regressing WTP-Q values, calculated according to Eq. (4), on the specified covariates for each scenario separately. Given that probability weighting was used to calculated expected QALY gain, the regression was run four separate times, once with no probability weighting and then with the three probability weighting specifications. In a second step, estimation results were used to predict WTP-Q values for each individual. Taking the mean of these individual predictions results in the final regression-based WTP-Q estimates.

4 $${WTP\_Q}_{scenario}=\frac{mean({WTP}_{scenario}*12)}{mean({E\left(Q\right)}_{scenario})}$$

5 $$\mathrm{ln}(WTP\_Q) =\,\alpha +{{\beta }_{1}scenarios+{ \beta }_{2}married+ \beta }_{3}\mathrm{ln}\left(income\right)+ {\beta }_{4}age+ {\beta }_{5}{age}^{2}+ {\beta }_{6}male {+ \beta }_{7}SAH+ {\beta }_{8}education + {\beta }_{9}employment+ {\beta }_{10}awareness+ {\beta }_{11 }country+ \varepsilon$$

A key benefit of using linear regression to predict WTP-Q is correcting for individual characteristics. Given the somewhat smaller sample sizes for some of the scenarios (e.g. death and high-risk) the respondent groups may not have identical characteristics. Therefore, if we wish to compare WTP estimates for each scenario, the prediction approach is arguably the most apt for our current purposes.

## Results

### Willingness-to-pay per scenario

First, summary statistics of raw WTP by scenario can be examined. This not only signals general willingness to pay for an EWS under different scenarios, but also provides first insight into the theoretical validity and plausibility of the questionnaire and respondents’ answers. Table 3 first of all shows that, for every scenario, 92% or more of respondents were willing to pay something (i.e. > 0) for an EWS. Moreover, from Table 3 it appears that the mean WTP for all other scenarios was higher than in the basic scenario, with all means lying between 17 and 24 Euros. For instance, an EWS would be valued about 39% higher when designed to prevent a catastrophic scenario, than in case of a ‘basic’ scenario. Given that there also were less protest answers for the catastrophic scenario, the aggregated value for an EWS in this case would be valued even higher than in a basic scenario. Standard deviations ranged between 21 and 28 euros dependent on the scenario, showing the considerable heterogeneity between responses, and suggesting that differences in means are surrounded with uncertainty. The scenario with the largest monthly mean WTP (€24.23) was the catastrophic scenario, where respondents were asked their WTP for a 40% reduction in risk (form 60%-20%) of immediate death. Finally, we present an approximation of an aggregate WTP per scenario across all six countries, using the same assumptions as Himmler and colleagues used—i.e. taking the median WTP, and assuming that 50% of households (excluding the percentage of protest answers) would be eligible to pay the additional tax (calculations shown in Appendix 1) [7]. These aggregate estimates range between 4.4 and 7.8 billion euros per year.

**Table 3:** Summary statistics monthly WTP by Scenario

Scenario	Mean (sd)	Median	Min	Max	% WTP > €0	Obs	Total in millions
Basic	17.35 (20.70)	8.50	0	97.75	95.1	2542	5069 m
Certainty	20.29 (24.07)	10.80	0	112.79	93.7	1402	6434 m
High Risk	20.25 (24.12)	10.80	0	107.96	96.3	616	6292 m
Death	17.76 (20.60)	9.57	0	97.56	95.9	542	5882 m
Equity	20.00 (23.39)	11.72	0	108.25	95.3	725	7118 m
Social exclusive	18.42 (23.26)	8.13	0	103.10	92.3	627	4395 m
Catastrophic	24.23 (28.11)	13.17	0	140.99	96.5	1141	7776 m

The regression on ln(WTP + 1)—shown in Table 4—highlights how each element of the scenarios (e.g. size of risk, health deterioration or death) affects WTP along with other key determinants, and shows face validity for several of the elements in question. The results show that a large risk reduction (e.g. 40% rather than 2%) was significantly correlated with a 15% higher WTP. Given that the large risk reduction is 20-fold the small risk reduction, it is fair to conclude that this increase in WTP is not proportional to changes in risk. We also see that certainty (risk dropping to 0%) was significantly correlated with a 10% higher WTP. Death being the outcome of the infectious disease (as opposed to a one year reduction in health) was correlated with a 20% increase in WTP. The social exclusive perspective (in which respondents themselves are not the beneficiaries of the EWS) had a limited negative, although insignificant, relationship with WTP (− 4%). The magnitude of the difference between ‘better’ and ‘worse’ health states (or ‘better’ and death health states), had no visible effect on WTP responses. With regards to demographic characteristics, we found that income had a positive effect on WTP while self-assessed health (EQ-5D score) had a negative effect on WTP.

**Table 4:** OLS regression on WTP

	ln(WTP + 1)	
	Estimates	Standard Error
Large risk (Ref. 40% reduction instead of 2%)	0.20*	0.01
Certain risk (Ref. Risk reduced to 0% instead of 2%/20%)	0.09*	0.01
Socially exclusive (Ref. Not socially exclusive)	− 0.06*	0.01
Outcome death (Ref. health deterioration for 1 year)	0.11*	0.02
Difference in health states	− 0.00	0.00
Ln(income)	0.14*	0.03
Age	− 0.07*	0.01
Age^2^	0.00*	0.00
Male	0.13*	0.04
EQ-5D score	− 0.07	0.19
Tertiary education^a^	0.06	0.04
Married	0.19*	0.04
Student^b^	0.09	0.12
Employed^b^	0.14	0.09
Retired^b^	0.15	0.11
Self-employed^b^	0.05	0.11
Unable to work^b^	− 0.30*	0.14
Unemployed^b^	0.03	0.12
Awareness of outbreaks^†^	0.02*	0.00
Data quality dummy	− 0.07	0.04
Denmark^c^	0.36*	0.08
Germany^c^	0.23*	0.07
Italy^c^	0.54*	0.07
Netherlands^c^	0.30*	0.07
UK^c^	0.08	0.07
Constant	1.96*	0.39
Observations	6291	
R2/R2 adjusted	0.166/0.163	
AIC	3847	

*P values < 0.05

^a^Base case: No tertiary education

^b^Base case: Homemaker, ^c^Base case: Hungary

^†^scored from 12 to 84 (12 questions with 7 levels)

We also found an effect of home country on WTP, using Hungary as the base-case. So even after controlling for many important individual characteristics, WTP varied significantly across countries. While we do not know what it exactly drive these differences, we hypothesise, as Himmler et al. in the context of the initial estimate of WTP for an EWS [7], that variation in cultural dimensions and trust in public institutions may contribute to them. Countries included in this study were in fact selected to provide a range of cultural perspectives, which were assessed using the three most relevant dimensions of Hofstede’s cultural dimensions: individualism vs collectivism, masculinity, and uncertainty avoidance [7, 34].

### WTP-Q estimates

The OLS regression shown in Eq. 5 allowed the isolation of the effects of different scenarios on log-transformed WTP-Q. The model was fit to both probability weighted and non-probability weighted WTP-Q estimates, so that comparisons between weighting approaches could be investigated. The results are displayed in Table 5. This regression-based approach used the same control variables (not displayed) as in Table 4, and resulted in coefficients similar to those shown in Table 4 across models.

**Table 5:** OLS regression on ln(WTP-Q) estimates

	No weighting^†^		TK^†^		Prelec^†^		GW^†^	
Predictors	Estimates	SE	Estimates	SE	Estimates	SE	Estimates	SE
Certainty scenario^a^	− 0.29*	0.01	− 0.36*	0.01	− 0.57*	0.01	− 0.37*	0.01
High risk scenario^a^	− 0.90*	0.02	− 0.55*	0.01	− 0.62*	0.02	− 0.58*	0.01
Death scenario^a^	− 1.04*	0.02	− 0.76*	0.02	− 0.86*	0.02	− 0.78*	0.02
Equity scenario^a^	− 0.30*	0.02	− 0.36*	0.01	− 0.58*	0.02	− 0.37*	0.01
Social exclusive scenario^a^	− 0.36*	0.02	− 0.40*	0.01	− 0.60*	0.02	− 0.41*	0.01
Catastrophic scenario^a^	− 1.05*	0.01	− 0.77*	0.01	− 0.87*	0.01	− 0.79*	0.01
Observations		6679		6679		6679		6679
R2/R2 adjusted		0.556/0.554		0.517/0.515		0.553/0.551		0.521/0.520
AIC		3960.893		1473.359		3261.685		1721.054

*P values < 0.05

N.B. Same control variables used as in Table 4

^a^Base case: Basic scenario, ^b^Base case: No secondary school diploma, ^c^Base case: Not working, ^d^ Base case: Hungary

^‡^UK value set used, ^†^scored from 12 to 84 (12 questions with 7 levels)

WTP-Q estimates only calculated where EQ was not equal to 0, so as to avoid infinite values

^†^No PW = no probability weighting. TK = functional form from Tversky and Kahneman [13] $w\left(p\right)= \frac{{p}^{\gamma }}{{{[p}^{\gamma }+{(1-p)}^{\gamma }]}^{\frac{1}{\gamma }}}$; P = functional form Prelec [27]:$w\left(p\right)=\mathrm{exp}(-(-{\mathrm{ln})}^{\alpha })$; GW = functional form Gonzalez and Wu^26^:$w\left(p\right)= \frac{{\delta p}^{\gamma }}{{{[\delta p}^{\gamma }+(1-p)]}^{\gamma }}$

All scenarios had a significant (negative) effect on WTP-Q in comparison to the base scenario. In this case it is the difference in magnitude that is the most interesting. We see that (without weighting) the high risk, death and catastrophic scenarios led to a WTP-Q estimate that was at least 90% lower than the WTP-Q estimate in the base-case, ceteris paribus. Certainty, equity, and social exclusive scenarios lead to WTP-Q that was around 30% lower.

While the linear regression estimates displayed in Table 5 provided us with the relevant information on the impact of scenarios and probability weighting on WTP-Q, Table 6 gives absolute values for the WTP for a health gain in the context of a pandemic, for each scenario, methodological approach, and probability weighting functional form.

**Table 6:** WTP-Q for each scenario calculated ratio of Means and Linear Regression (LR) Prediction

	Basic		Certainty		High risk		Death		Equity		Social exclusion		Catastrophic	
	Ratio of means	LR	Ratio of means	LR	Ratio of means	LR	Ratio of means	LR	Ratio of means	LR	Ratio of means	LR	Ratio of means	LR
No PW^a^	30,488	30,238	17,824	17,128	1778	1743	364	381	17,573	16,545	16,181	15,657	25	25
TK^a^	17,384	17,242	7064	6789	2826	2768	207	217	6965	6558	6413	6206	39	40
P^a^	21,301	21,126	4601	4422	3213	3148	254	266	4536	4271	4177	4042	45	46
GW^a^	18,217	18,068	7411	7121	2655	2602	217	228	7306	6879	6727	6510	37	38
N	2542	2237	1402	1229	616	544	542	476	725	634	627	551	1141	1008

^a^No PW = no probability weighting. TK = functional form from Tversky and Kahneman [13]: $w\left(p\right)= \frac{{p}^{\gamma }}{{{[p}^{\gamma }+{(1-p)}^{\gamma }]}^{\frac{1}{\gamma }}}$; P = functional form Prelec [27]:

$w\left(p\right)=\mathrm{exp}(-(-{\mathrm{ln})}^{\alpha })$; GW = functional form Gonzalez and W [26]: $w\left(p\right)= \frac{{\delta p}^{\gamma }}{{{[\delta p}^{\gamma }+(1-p)]}^{\gamma }}$. LR = linear regression prediction

Results varied significantly depending on the scenario and the used weighting approach. For example, in the Basic scenario, the ratio of means approach (weighted using TK form) led to a WTP-Q of €17,000. Unweighted, the WTP-Q was €30,000 in the Basic scenario. The linear regression estimates generally were slightly lower compared to the ratio of means estimates (expect in the Death scenario). Looking at the scenarios, WTP-Q ranged from €30,000 (basic scenario) to €25 (catastrophic scenario) across methods and weighting approaches. Even when considering a single methodological approach and probability-weighting functional form differences in WTP per QALY across scenarios were still large.

Scenarios in which health gains were largest, such as where death was the result of infection, and where reduction in risk was greatest, had the lowest WTP-Q estimates. Certainty, equity and social exclusive scenarios, which all had the same reduction in risk and health gains, resulted in similar estimates, in the area of €16,000-€19,000 (unweighted).

## Discussion

### Summary of results

The need for and potential benefits of EWSs for infectious disease and foodborne outbreaks have rarely been more apparent than in recent times, and thus research on investment into EWSs is of relevance. The aim of this paper was to add to the available literature by providing estimates of WTP for EWSs as well as WTP-Q in the context of infectious disease outbreaks, both for seven different scenarios, using a representative sample at the European level. These scenarios were chosen to investigate the impact of changes in risk reduction, the context in which health is gained (decrease in health or death) and whether a SIP or social exclusive perspective is taken. In doing so, some additional methodological issues came to light, specifically the (in)sensitivity to scale of WTP and the subsequent effects on WTP-Q.

Our design allowed to consider both directly WTP for an EWS in different contexts as well as calculating the implied WTP per Q estimates. In that respect, it is important to note that WTP values are the estimates that were directly elicited via the questionnaire. WTP-Q estimates were calculated on the basis of the indicated WTP and our calculations of the implied health gains (in terms of QALYs). Using the payment vehicle of increased taxes, we found that WTP was driven, at least in part by the size and certainty of the risk presented to respondents, the outcome of the disease in question (e.g. death), and various demographic variables such as income and age. Given that all coefficients moved in the expected direction, our direct WTP results can be considered to be ‘theoretically valid’ [15], although not near proportional. The fact that the magnitude of the differences between the better and worse health state did not significantly affect the WTP answers (Table 4) may indicate that respondents focused more on elements that were explicitly presented to them in the WTP questions.

Using these direct WTP results to estimate aggregate values of an EWS, we found total values for an EWS between 4.4 and 7.8 billion euros per year for the six European countries covered in the questionnaire—the largest value in relation to the catastrophic scenario and the smallest when presenting the social exclusive scenario (a difference of 56%).

When translating the direct WTP estimates into WTP-Q estimates, the range of estimates produced was large, especially due to the fact that changes in direct WTP were not (near) proportional to changes in risk reductions and, therefore, implied health gains. Therefore, the practical plausibility of the resulting WTP-Q estimates may be questioned. Lack of scope sensitivity has been observed before in related studies in health economics [15, 35, 36], but also in other fields like ecological economics [37, 38]. This underlines the importance of further research into the optimal designs for contingent valuation in the context of collectively financed healthcare and health gains and the validity of obtained estimates. Comparing the performance of contingent valuation with alternative methods like discrete choice experiments is also encouraged in this context.

WTP-Q was estimated using two separate approaches (ratios of means and linear regression), and three approaches for probability weighting. This showcased the effects of different methodological approaches on the WTP-Q estimates. Given that the linear regression method controls for demographic variables that could affect WTP-Q, we focus on those results in this discussion section, specifically those probability weighted with the Tversky and Kahneman function. Regarding the results, an initial WTP-Q value, for the basic scenario was estimated at €17,400. This can be interpreted as a threshold for investment per QALY into an EWS for infectious disease outbreaks. Note that these WTP-Q estimates may include broader benefits related to the EWS that respondents could consider and value when providing their WTP, and, therefore, could be seen as a ‘WTP for a QALY + ’. This could concern intangible benefits, like feelings of safety, but also cost-savings—respondents may for instance be willing to pay more money now to avoid a reduction in income later. Given that this questionnaire was answered before the COVID-19 outbreak, this may have been less of a consideration than post-March 2020, but still it cannot be excluded. This may also affect some scenarios more than others. WTP responses may also include reflect expectations about income or productivity in particular situations that would normally be captured separately in an economic evaluation, highlighting the need to avoid double-counting.

The base-case was only one of seven scenarios examined in this paper. When comparing the results from these seven scenarios, the implied WTP-Q estimates varied very considerably. This was not only the case when comparing different methodological approaches to estimating WTP-Q from WTP data and implied health gains in the different scenarios, but especially related to the fact that respondents’ WTP was clearly not proportional to the magnitude of the implied health gains. This holds both in terms of the size of the risk reduction and in terms of the size of the difference between health states. We, therefore, found that differences between the obtained WTP-Q estimates, in which WTP is divided by expected health gain per scenario, were predominantly driven by this denominator, rather than the differences in WTP responses themselves. This lack of sensitivity (at least in relation to implied health gains) may be related to the fact that we were valuing a system for disease surveillance, which is preventive (rather than curative) in nature, and is concerned with hypothetical, future emergencies. Perhaps that somewhat drew attention away from the size of the health gains on offer, although sensitivity to scope is a more commonly observed phenomenon in this context [17, 35, 36]. It would be interesting to see the same scenarios attached to a curative intervention when eliciting WTP per QALY.

### Context of study results

The main results of this paper are in line with previous work on the topic [14, 15, 39]. ‘Raw’ WTP results are theoretically valid, and move in the directions expected; individuals are willing to pay more for the certainty and death scenarios than the basic scenario, although only results for the certainty scenario were significant. These estimates are, however, far from theoretically plausible, given their lack of near-proportionality in relation to the implied size of the health gains. This is worrisome, given the consequences of this combination on the subsequent WTP-Q measures. When WTP estimates lack sensitivity to scale, this results in WTP-Q estimates that are highly variable and hard to consider theoretically valid when comparing across scenarios. For example, while the non-probability weighted basic scenario estimate of €30,000 per QALY is in line with previous estimates [14, 15, 39], in the same sample the estimates from the certainty and catastrophic scenarios result in much lower WTP-Q estimates. Even though low WTP-Q estimates have been reported before—where QoL improving interventions were valued lower than life-extending interventions [40]—the fact that such a wide range of values was found in our results raises the issue of which value is ‘correct’.

If WTP were sufficiently sensitive to scale, then the WTP-Q estimates should remain relatively similar across scenarios. Given that this is not the case, and it appears unlikely that the resulting WTP-Q estimates reflect ‘true valuations’ of QALY gains in different contexts, this emphasises the difficulty of finding a unique, universally valid WTP-Q estimate. It is clear that methodological assumptions made, such as whether to use probability weighting (and even which weighting function to use), the framing of WTP questions, possible anchoring effects, and the approach used to estimate WTP-Q, strongly affect the outcomes of the research.

### Limitations and strengths

There are limitations to our study. First, while anchoring may be responsible for later WTP estimates being close in magnitude to the WTP for the basic scenario, it is not possible to fully empirically investigate this using the data available—there was no change in ordering of questions for any respondents. That being said, in auxiliary analysis (Appendix 1) we found that the higher responses to the initial question posed to respondents, the greater the WTP for the next questions. However, separating anchoring effects from other issues such as ordering effects or simply individual preferences is not possible. It is advised when collecting data for the estimation of WTP-Q to provide multiple orderings of scenarios so that anchoring can be investigated.

Second, while our sample is representative of the population for the basic scenario with regards to age and gender, it was not aimed to be representative in terms of other factors (due to feasibility) which may impact WTP-Q responses. Furthermore, it is likely that when respondents are randomly assigned to the other six scenario questions, that the sample will not be fully representative of each country’s population. However, by predicting WTP-Q using linear regression models many of these demographic characteristics are accounted for. Fourth, life-tables were used to estimate expected health gains when death was an outcome in a specific scenario. It may, however, be that life-expectancy from the Human Mortality Database is different from the subjective life-expectancy an individual had in mind when filling in the questionnaire (for example respondents may over-estimate their life-expectancy) [41].

Third, the data used were collected via online questionnaires in March of 2018, when there was no global pandemic or even knowledge of COVID-19. Given the now more obvious need for EWSs, the WTP for an EWS is higher today. This was also shown in a recent study, re-fielding the survey in April 2020 [42].

Future research would also benefit from further investigation into questionnaire design when eliciting WTP-Q, and into the effects that using such estimates of cost-effectiveness thresholds may have on policy. For example, what precisely is included in the WTP estimates is currently somewhat of a black box—it would be useful to try and disaggregate some of the elements that may be included in the valuation (e.g. QALYs, safety, cost-savings, productivity) in future studies. Furthermore, it is possible that by carrying out the questionnaire online, there was less engagement with the questionnaire and (hence) a lower quality of the answers than if the questionnaire had been completed in person. Carrying out (at least a percentage of) in person questionnaires alongside the online questionnaire would have highlighted any systematic differences in responses [43].

Fourth, we present results obtained in a study with a particular design, using taxes as payment vehicle and alluding to a collectively financed healthcare system. It is important to emphasize that in this paper, therefore, rather than focusing on own valuations of own health through direct payments, we here valued health gains from a societal perspective (see e.g. [17]). While this comes with disadvantages, it is deemed most relevant in the context of collectively funded healthcare systems, like common in Europe. Hence, using an increase in taxes as the payment vehicle, therefore, for an early warning system was considered plausible and appropriate, also to allow observing relevant valuations of health gains in specific groups (like children) and the inclusion of the notion that all citizens will have to pay into the system. Indeed, this may be considered relevant in the context of policy decisions, like those regarding early warning systems on a (supra)national level, but nonetheless also results in valuations that do not necessarily (solely) reflect own valuations of own health gains. Moreover, using this payment vehicle may have invoked responses related to increases in taxes in a general sense, arguably impacting our results, for instance through more protest answers. Moreover, investigating whether using taxes as payment vehicle is sufficiently incentive compatible, also given its collective nature, is something that should be investigated further.

There were also strengths to this study, a key one being that data were collected from six European countries, which were chosen specifically to both cover different ends of the spectrum with regards to three of Hofstede’s cultural dimensions: individualism vs collectivism, masculinity, and uncertainty avoidance [34] and to cover a wide economic range. This may enhance the generalisability (to the European level) of our results. Furthermore, the use of a two-step payment scale approach and an open question to elicit WTP leads to more precise estimates of WTP, as it combines two (linked) elicitation questions [44–46]. Additionally, the use of two separate estimation approaches, provides a thorough overview of the impact of methodological choices on WTP-Q estimation.

### Conclusions

This study has provided estimates of the monetary value of a health gain in the context of a pandemic under seven scenarios which differ in terms of outcome, risk reduction and those affected. The effects of probability weighting were also investigated. While the (probability weighted) WTP-Q for the basic scenario lies somewhere within an expected range for previous estimates of WTP-Q in the literature (€17,000), the large variation in estimates for the six other scenarios is concerning if this threshold were to be used in the decision-making context. That being said, it is clear from the aggregate estimates of WTP that an EWS is an intervention that is valued in European populations. This is encouraging when we consider than EWS interventions are one of the ways to avoid repeat pandemics and their negative consequences on health and welfare.

WTP-Q estimates also represent first estimates of a possible cost-effectiveness threshold for the reimbursement of preventive interventions, specifically those that generate feelings of safety along with QALY gains. However, they also raise several questions around the choice of questionnaire design when trying to elicit such values, the reliability of using contingent valuation to elicit WTP for a QALY estimates, and whether using an ‘augmented’ WTP-Q estimate brings value or overcomplicates an already complex area of decision-making. Given that these are initial estimates with much uncertainty, we recommend, at the very least, that these results be considered with caution, and that preferably research should be done into how intervention specific thresholds (that may cover outcomes outside of the QALY) can or should fit into decision making around public health.

## Appendix 1

See Tables 7, 8, 9, 10, 11 and 12.

**Table 7:** Age and gender distribution of analysis sample (*n* = 2920) compared to population sampling quotas used by the sampling agency

		DK		GER		HUN		IT		NL		UK	
		Sample (%)	Quota	Sample (%)	Quota	Sample (%)	Quota	Sample (%)	Quota	Sample (%)	Quota	Sample (%)	Quota
Male	18–24	36 (7.5)	6.4	12 (2.5)	6.0	24 (5.1)	6.7	36 (7.3)	5.4	32 (6.6)	6.4	33 (6.4)	7.0
	25–34	53 (11.1)	8.6	51 (10.6)	8.5	55 (11.6)	9.3	45 (9.2)	9.1	46 (9.4)	8.7	55 (10.7)	9.9
	35–44	47 (9.9)	10.3	50 (10.3)	10.3	53 (11.2)	11.4	43 (8.8)	11.7	46 (9.4)	10.9	62 (12.1)	10.1
	45–54	47 (9.9)	10.5	50 (10.3)	10.9	45 (9.5)	8.6	45 (9.2)	10.5	52 (10.7)	10.9	51 (10.0)	10.1
	55–65	56 (11.8)	14.2	63 (13.0)	14.4	54 (11.4)	13.0	67 (13.7)	12.9	76 (15.6)	13.4	49 (9.6)	12.4
Female	18–24	52 (10.9)	6.2	38 (7.9)	5.6	32 (6.7)	5.8	36 (7.4)	5.1	35 (7.2)	6.2	37 (7.2)	6.8
	25–34	43 (9.1)	6.6	53 (11.0)	8.5	58 (12.2)	9.1	55 (11.3)	8.9	49 (10.1)	8.7	48 (9.4)	9.9
	35–44	41 (8.6)	10.3	58 (12.0)	10.1	53 (11.2)	11.2	63 (12.9)	11.6	46 (9.4)	10.7	46 (9.0)	10.5
	45–54	48 (10.1)	10.3	51 (10.6)	10.7	50 (10.5)	8.8	46 (9.4)	10.8	42 (8.6)	10.7	63 (12.3)	10.3
	55–65	52 (10.9)	14.6	57 (11.8)	15.0	51 (10.7)	16.0	52 (10.7)	14.0	63 (12.9)	13.4	68 (13.3)	13.0
Total		475	1	483	1	475	1	488	1	487	1	512	1

Quotas relate to the population of individuals aged between 18 and 65, the inclusion criteria in the study

**Table 8:** Expected answers for health state valuation

Statement	# Responses	% of sample
Direct comparison^a^ Health State 1 > Health state 2	220	92.99
Health State 1 > Health State 2—Consistently in both tasks^b^	2267	72.20
Inconsistent answers for Health State 1 > Health State 2	749	23.85
Health State 1 > Death	2105	67.04
Health State 2 > Death	1970	62.74
All answers as expected^c^ (HS1 > HS2, HS1 > Death, HS2 > Death)	1422	45.29

^a^Second figure in Appendix 2. Used as exclusion criteria due to data quality (Table 1)

^b^Rated health state 1 better in both tasks shown in Appendix 2

^c^Used as dummy variable for data quality in the regressions

**Table 9:** Probability weighting and parameter estimates

Non weighted probabilities for each scenario	Weighted probabilities		
	TK^a^ γ = 0.674 for losses	P^a^ α = 0.533	GW^a^ δ = 0.84, γ = 0.65 for losses
Basic scenario			
0.04	0.10	0.15	0.10
0.02	0.07	0.13	0.06
Certainty			
0.04	0.10	0.15	0.10
0.00	0.00	0.00	0.00
High risk			
0.60	0.51	0.50	0.52
0.20	0.26	0.13	0.25
Death			
0.04	0.10	0.15	0.10
0.02	0.70	0.13	0.06
Equity			
0.04	0.10	0.15	0.10
0.00	0.00	0.00	0.00
Social exclusive			
0.04	0.10	0.15	0.10
0.00	0.00	0.00	0.00
Catastrophic			
0.60	0.51	0.50	0.52
0.20	0.26	0.13	0.25

^a^TK = functional form from Tversky and Kahneman [13]: $w\left(p\right)= \frac{{p}^{\gamma }}{{{[p}^{\gamma }+{(1-p)}^{\gamma }]}^{\frac{1}{\gamma }}}$

P = functional form Prelec [27]: $w\left(p\right)=\mathrm{exp}(-(-{\mathrm{ln})}^{\alpha })$

GW = functional form Gonzalez and W [26]:$w\left(p\right)= \frac{{\delta p}^{\gamma }}{{{[\delta p}^{\gamma }+(1-p)]}^{\gamma }}$

**Table 10:** Effect of initial EWS answer on Basic Scenario answer

	Basic Scenario answer			
Predictors	Estimates	Std. Error	Estimates	Std. Error
Initial answer (reported elsewhere)^†^	0.86*	0.01	0.86*	0.01
Income	0.63	0.34	0.62	0.34
Age	− 0.14*	0.02	− 0.14*	0.02
Male	1.07*	0.51	1.05*	0.51
Tertiary education	− 0.91	0.53	− 0.92	0.53
Married	0.72	0.56	0.75	0.56
Student^a^	− 1.36	1.45	− 1.35	1.45
Employed^a^	− 0.05	1.12	− 0.01	1.13
Retired	1.94	1.39	1.96	1.40
Self− employed	0.15	1.36	0.18	1.37
Unable to work	− 1.44	1.77	− 1.37	1.77
Unemployed	0.65	1.47	0.64	1.47
Denmark^b^	0.17	0.97	0.08	0.97
Germany^b^	− 0.59	0.89	− 0.70	0.90
Italy^b^	− 0.38	0.92	− 0.49	0.92
Netherlands^b^	− 1.98*	0.93	− 2.10*	0.94
UK^b^	− 0.72	0.87	− 0.81	0.87
Answer Pattern 2A-3B^a^			0.45	0.68
Answer Pattern 2B-3C^a^			− 0.37	0.70
Answer Pattern 2C-3C^a^			0.32	0.72
Constant	4.64	2.72	4.67	2.75
Observations	2145		2145	
R2 / R2 adjusted	0.659/0.656		0.659/0.656	
AIC	16,615		16,619	

**P* values < 0.05

^†^Himmler et al. [7]

^a^Base case: Answer patterns 2A-3A (see Fig. 1)

^b^Base case: Homemaker

^c^Base case: Hungary

**Table 11:** Effect of Basic Scenario answer on following answer

	Second scenario answer			
Predictors	Estimates	Std. Error	Estimates	Std. Error
Initial answer (reported elsewhere)^†^	0.91*	0.01	0.92*	0.01
Income	0.90*	0.34	0.94*	0.33
Age	− 0.02	0.02	− 0.02	0.02
Male	0.88	0.50	0.89	0.50
Tertiary education	0.61	0.51	0.59	0.51
Married	0.58	0.54	0.53	0.54
Student^a^	3.92	1.41*	3.93*	1.40
Employed^a^	− 0.22	1.09	− 0.27	1.09
Retired	− 0.69	1.36	− 0.75	1.35
Self-employed	− 1.12	1.33	− 1.11	1.33
Unable to work	− 0.04	1.70	− 0.19	1.70
Unemployed	0.51	1.43	0.51	1.43
Denmark^c^	0.24	0.94	0.46	0.95
Germany^c^	− 0.64	0.87	− 0.30	0.87
Italy^c^	1.07	0.88	1.36	0.89
Netherlands^c^	− 1.23	0.89	− 0.88	0.90
UK^c^	0.15	0.84	0.45	0.84
Answer Pattern 2A–3B^a^			− 0.85	0.66
Answer Pattern 2B–3C^a^			1.38*	0.67
Answer Pattern 2C–3C^a^			− 1.44*	0.69
Constant	− 3.58	2.65	− 3.88	2.66
Observations		2181		2181
R2 / R2 adjusted		0.736/0.734		0.739/0.736
AIC		16,778		16,766

**P* values < 0.05

^†^Himmler et al. [7]

^a^Base case: Answer patterns 2A-3A (see Fig. 1)

^b^Base case: Homemaker

^c^Base case: Hungary

**Table 12:** Aggregate WTP

Scenario	Median WTP in € per month^1^	No. of household in million^2^	% Protest zeros^3^	% HH paying tax	Total in millions
Basic	8.5	108.81 m	8.66%	50	5069 m
Certainty	10.8	108.81 m	8.75%	50	6434 m
High risk	10.8	108.81 m	10.76%	50	6292 m
Death	9.57	108.81 m	5.86%	50	5882 m
Equity	11.72	108.81 m	6.97	50	7118 m
Social exclusive	8.13	108.81 m	17.19	50	4395 m
Catastrophic	13.17	108.81 m	9.56	50	7776 m

^1^Based on Table 3

^2^Assumption based on the share of households with income taxpayer who are eligible for additional taxation

^3^Based on Table 1

## Appendix 3

### Example of description and resulting questions for scenario 1

#### [Introduction presented before module 3]

##### 1A. [Introduction]

Suppose 100,000 people in your country are in the health state that you previously selected as the better one. Due to a virus, these people face a 4% risk of becoming infected with a virus within the next three months. It is not known who belongs to this group. The health of those infected deteriorates to the health state you just selected as the worse one. They will remain in the worse health state for a year, and then return to the better health state.

[explanation of risk with and without an early warning system].

To illustrate what the percentage risk means, click on one of the squares in the picture below [show picture with only green squares].

[The following text and picture will appear when respondent clicks on one of the squares].

In a group of 100 people, 4 in 100 (4%) will become sick and 96 will not become sick. To get a sense of what an integrated early warning system would mean for a group of 100 people, please click again on one of the squares in the picture below.

The chance that the square you selected turned red was 4%

Imagine that the risk of being infected by this virus can be reduced from 4 to 2% through an international, integrated early warning system that helps contain and mitigate [mouse cursor] the spread of the virus. However, establishing and maintaining such a system is not without costs. Suppose the funding would take place through taxation, paid by all eligible people in your country (above 18 years of age). This taxation would be collected through monthly instalments for the duration of one year.

To illustrate what the percentage risk means, click on one of the squares in the picture below [The following text and picture will appear].

[The following text and picture will appear when respondent clicks on one of the squares].

In a group of 100 people, 2 in 100 means (2%) will become sick and 98 will not become sick.

The chance that the square you selected turned red was 2%

##### 1B [Payment scale]

Suppose you would have to pay this monthly instalment starting now. Please consider the amounts on the scale below, from left to right, and select the amounts you would definitely be willing to pay per month for establishing and maintaining this integrated early warning system. This amount would then be a taxation paid by all eligible people in your country. Please keep in mind your ability to pay (your net monthly household income).

[Save selected column as a variable. If € 0 is selected, proceed to 1D. If ‘more’ is selected, proceed to 1E. Otherwise, save selected amount as ‘X’ (for later use in 1G) and proceed to 1C.]

##### 1C. [Payment scale]

Now consider the same amounts below, now from right to left, and select the amounts you would definitely be willing to not pay per month for establishing and maintaining this integrated early warning system. This amount would then be a taxation paid by all eligible people in your country. Please keep in mind your ability to pay (your net monthly household income).

[Save selected column as a variable. If’more’ is selected, proceed to 1F. Otherwise, save selected amount as ‘Y’ (for later use in 1G) and proceed to 1G.]

##### 1D. [Follow-up question if 1B = € 0]

You have indicated that the maximum amount you would be willing to pay to establish and maintain an integrated early warning system is € 0.

Please indicate below, the reason behind this preference.

- Having an integrated early warning system is not worth more than € 0 to me
- I am unable to pay more than € 0
- Government should pay for this
- Other reason, namely ______________ (please specify; open text field, required if option is selected)

##### 1E. [Follow-up question if respondent indicates ‘more’ in questions 1B; if possible, on the same screen]

You have indicated that you would be willing to pay more than the maximum amount presented on the scale for establishing and maintaining an integrated early warning system, **which decreases your risk of becoming infected from 4 to 2%**. Please indicate in the box below the maximum amount that **you would be willing to pay** per month. This amount would then be a taxation paid by all eligible people in your country. Please keep in mind your ability to pay (your net monthly household income).

**Table:**

€	

##### 1F. [Follow-up question if respondent indicates ‘more’ in questions 1C; if possible on the same screen]

Please indicate in the box below the maximum amount that **you would not be willing to pay** for establishing and maintaining an integrated early warning system, **which decreases your risk of becoming infected from 4 to 2%.** Please keep in mind your ability to pay (your net monthly household income).

**Table:**

€	

[Save indicated amount as ‘Y3’ (for later use in 1G) and proceed to question 1G].

##### 1G. [Open-ended question; on new screen]

You have indicated that **you would definitely pay €[insert X]** per month and that **you would definitely not pay €[insert Y]** per month for establishing and maintaining an integrated early warning system, **which decreases your risk of becoming infected from 4 to 2%**. Please indicate in the box below the amount between €[insert X] and €[insert Y] that is closest to the maximum that you would be willing to pay per month. This amount would then be a taxation paid by all eligible people in your country. Please keep in mind your ability to pay (your net monthly household income).

**Table:**

€	
